# Supplementary material for: The Combination of ATM and Chk1 Inhibitors Induces Synthetic Lethality in Colorectal Cancer Cells
Source: Cancers (Basel). 2023 Jan 25;15(3):735. doi: 10.3390/cancers15030735 (PMC9913148; doi:10.3390/cancers15030735)
Supplement: Supplementary file 1 [file cancers-15-00735-s001.zip › cancers-2186909-supplementary.pdf]

Supplementary Materials

The combination of ATM and Chk1 inhibitors induces synthetic lethality in colorectal cancer cells

Yuri Tozaki, Hiromasa Aoki, Rina Kato, Kohki Toriuchi, Saki Sugino, Yasumichi Inoue, Hidetoshi Hayashi, Mineyoshi Aoyama

Components
Seven Supplemental Figures
Three Supplemental Tables

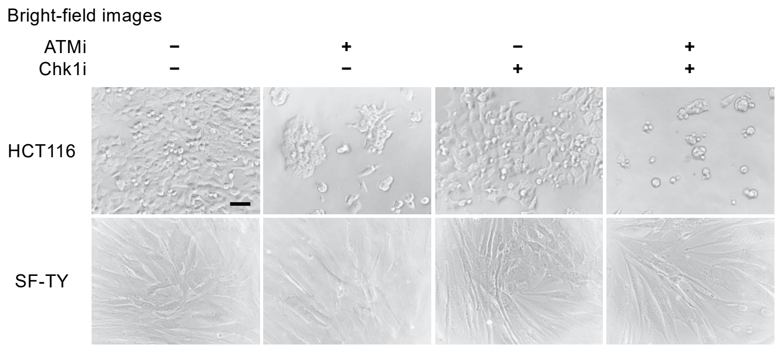

Figure S1. Bright-field images of drug-treated cells
Representative bright-field images of HCT116 and SF-TY cells after 48 h of treatment with the ATMi and/or Chk1i. Scale bar = 50 μm.

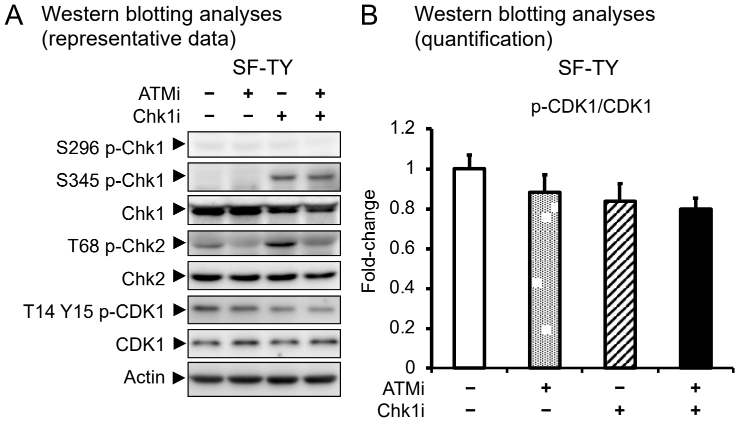

Figure S2. Phosphorylation of Chk1, Chk2, and CDK1 during drug treatment in SF-TY cells
(A) Western blotting analysis of Chk1, phospho-Chk1 (S296 and S345), Chk2, phospho-Chk2 (T68), CDK1, and phospho-CDK1 (T14 and T15) in SF-TY cells treated with ATMi (10 μM), Chk1i (0.03 μM), or both drugs for 6 h. (B) Quantification of the amount of each protein as determined by Western blotting in three cancer cell lines.

Protein levels were normalized to actin levels. Data are presented as mean ± SE ($n = 3$; ‡$p < 0.05$; Tukey's multiple comparison test). Uncropped versions of the immunoblotting data are shown in Supplementary Figure S3A. The relative intensity of each band in the immunoblots is shown in Supplementary Figure S3B.

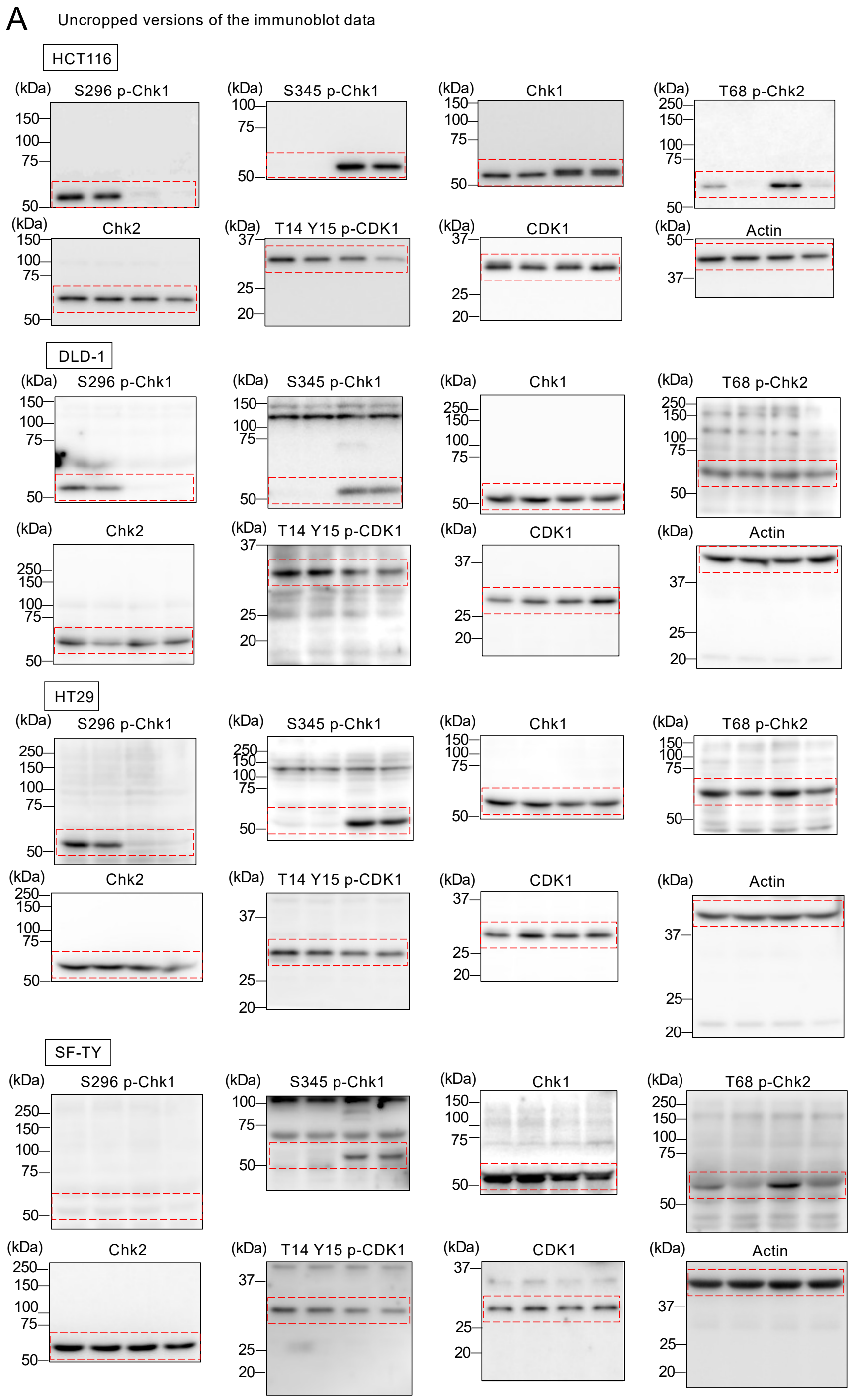

B Relative intensity of each band

	HCT116 cells				DLD-1 cells				HT29 cells				SF-TY cells			
	Control	ATMi	Chk1i	Combination	Control	ATMi	Chk1i	Combination	Control	ATMi	Chk1i	Combination	Control	ATMi	Chk1i	Combination
S296 p-Chk1/Actin	2.843	2.607	0.337	0.052	1.166	0.687	0.036	0.00	1.622	1.170	0.446	0.238	0.409	0.517	0.370	0.242
S345 p-Chk1/Actin	0.008	0.022	1.200	1.271	0.071	0.028	0.827	0.679	0.063	0.077	1.171	1.182	0.987	1.595	4.489	4.407
Chk1/Actin	0.485	0.473	0.704	0.878	1.244	1.092	0.995	0.9379	1.489	1.240	0.928	1.011	0.973	0.943	0.626	0.573
T68 p-Chk2/Actin	0.224	0.077	0.717	0.000	0.983	0.779	0.906	0.673	1.236	0.732	1.200	0.926	5.058	3.298	6.538	5.182
Chk2/Actin	0.962	1.021	0.977	0.935	1.020	0.619	0.905	0.747	1.193	1.218	1.030	0.759	1.181	0.953	0.974	0.832
T14 Y15 p-CDK1/Actin	0.895	0.744	0.689	0.467	1.950	1.482	1.068	1.012	1.216	0.874	0.619	0.606	4.757	4.264	3.245	3.036
CDK1/Actin	0.919	0.797	0.895	1.337	0.597	0.713	0.754	0.935	0.746	0.932	0.709	0.773	0.410	0.445	0.388	0.495

Figure S3. Supplemental information for Western blotting analyses
(A) Uncropped versions of the immunoblots shown in Figure 2 and Supplementary Figure S2. (B) Relative intensity of each band of the immunoblots shown in Figure 2 and Supplementary Figure S2.

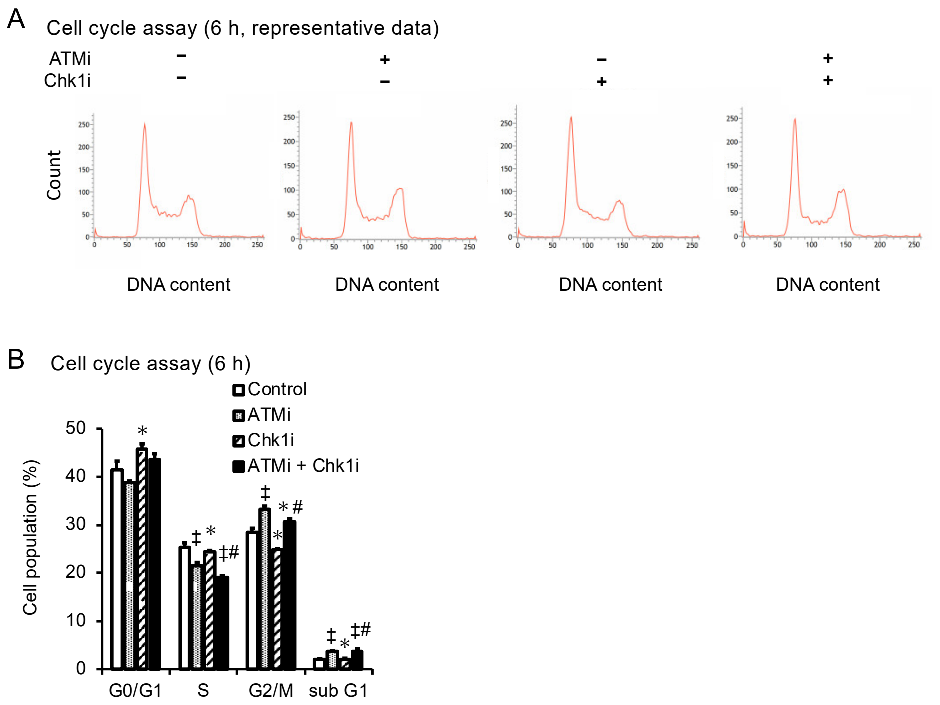

Figure S4. Analysis of cell cycle progression of cancer and normal cells under combined ATMi and Chk1i treatment for 6 h

(A) Representative results of assays of cell cycle progression of HCT116 cells treated with ATMi (10 μM), Chk1i (0.03 μM), or both drugs for 6 h, as determined by flow cytometry using PI staining. (B) Percentage of HCT116 cells in each cell cycle phase, as determined by PI staining. Data are presented as mean ± SE ($n = 3$; ‡$p < 0.05$, vs. control; *$p < 0.05$, vs. ATMi group, #$p < 0.05$, vs. Chk1i group; Tukey's multiple

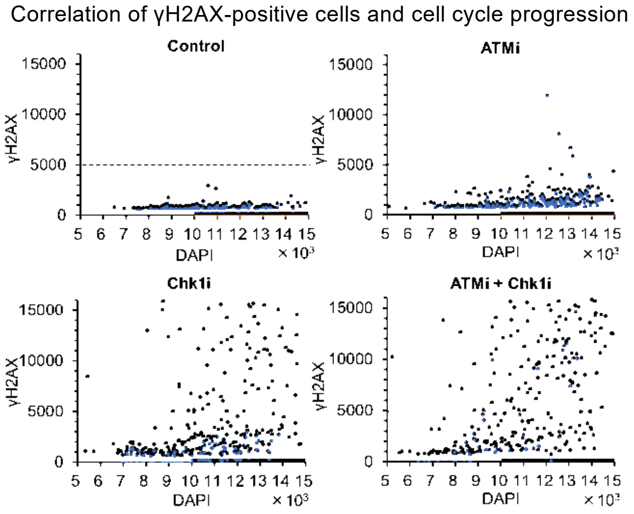

comparison test).

Figure S5. Analysis of the relationship between γH2AX-positive cells and cell cycle progression in cancer cells

Fluorescence intensity of γH2AX plotted against that of DAPI (300 cells in each group).

Based on the histogram of fluorescence intensity of DAPI in the control group, areas corresponding to the S and G2/M phases are indicated by orange bars.

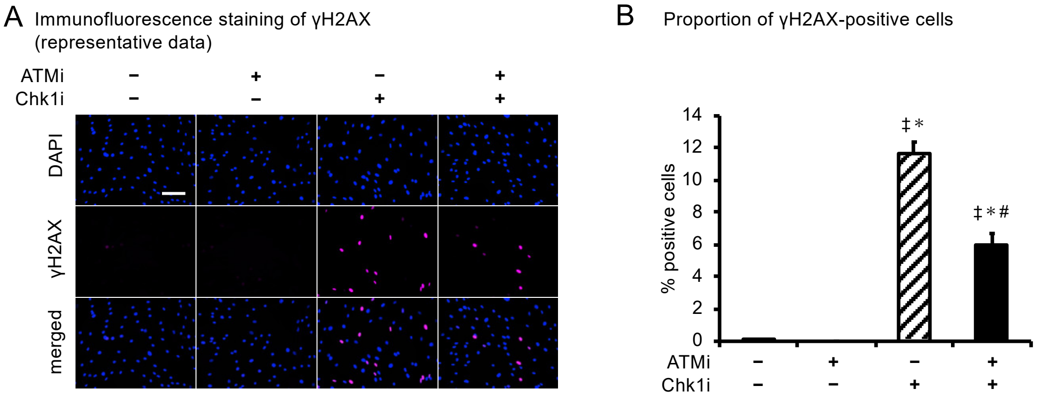

Figure S6. Analysis of DNA damage in normal cells under combined ATMi and Chk1i treatment

(A) Immunofluorescence staining of γH2AX (pink) in SF-TY cells treated with ATMi (10 μM), Chk1i (0.03 μM), or both drugs for 48 h. DAPI = blue. Scale bar = 100 μm. (B) Ratio (%) of γH2AX-positive SF-TY cells. Data are presented as mean ± SE ($n = 6$; 6 fields/well; ‡$p < 0.05$, vs. control; *$p < 0.05$, vs. ATMi group, #$p < 0.05$, vs. Chk1i group; Tukey's multiple comparison test).

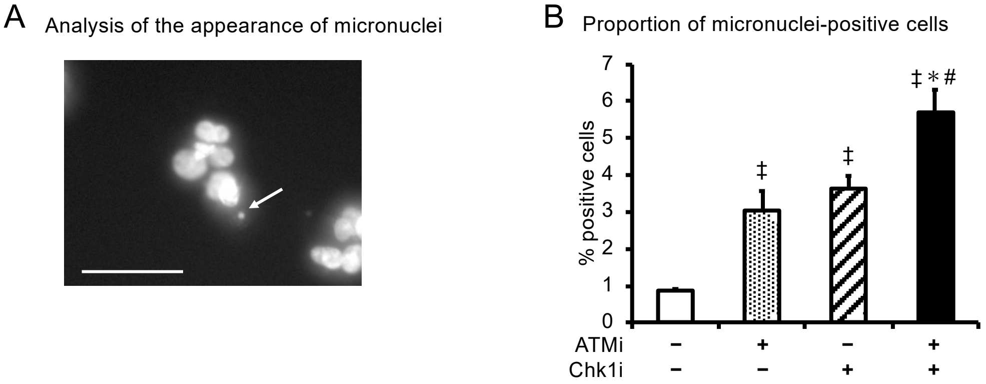

Figure S7. Analysis of the appearance of micronuclei in cells treated with the ATMi and Chk1i combination

(A) Representative image of micronuclei in HCT116 cells treated with the ATMi (10 μM) and Chk1i (0.03 μM) combination. Cells associated with micronuclei were detected using Harmony software. (B) Ratio (%) of micronuclei-positive HCT116 cells. Data are presented as mean ± SE ($n = 6$; 6 fields/well; ‡$p < 0.05$, vs. control; *$p < 0.05$, vs. ATMi group, #$p < 0.05$, vs. Chk1i group; Tukey's multiple comparison test).

Table S1. Antibodies used in Western blotting analyses

Target	Source	Catalog number	Species	Dilution
Phospho-Chk1 (S296)	CST	2349	Rabbit	1:1000
Phospho-Chk1 (S345)	CST	2348	Rabbit	1:1000
Chk1	CST	2360	Mouse	1:1000
Phospho-Chk2 (T68)	CST	2197	Rabbit	1:1000
Chk2	CST	6334	Rabbit	1:1000
Phospho-CDK1 (cdc2) (T14, Y15)	Invitrogen	44-686G	Mouse	1:1000
CDK1 (cdc2)	CST	9116	Mouse	1:1000
Actin	Sigma	A5060	Rabbit	1:1000

CST, Cell Signaling Technology.

Table S2. IC_{50} values of the ATMi and Chk1i

IC_{50}	ATMi (μM)	CHK1i (μM)
HCT116	18.4	2.79
DLD-1	26.5	0.56
HT29	8.91	24.2

Table S3. CI values for HCT116, DLD-1, HT29, and SF-TY cells

Cell type	Value	ATMi 10 μM + Chk1i 0.03 μM	ATMi 10 μM + Chk1i 0.1 μM	ATMi 10 μM + Chk1i 0.3 μM	ATMi 10 μM + Chk1i 1 μM
HCT116	CI	0.63	0.60	0.68	0.62
	FA	0.44	0.49	0.49	0.58
		ATMi 10 μM + Chk1i 0.03 μM	ATMi 10 μM + Chk1i 0.1 μM	ATMi 10 μM + Chk1i 0.3 μM	ATMi 10 μM + Chk1i 1 μM
DLD-1	CI	0.29	0.29	0.39	0.62
	FA	0.66	0.71	0.71	0.74
		ATMi 3 μM + Chk1i 0.3 nM	ATMi 3 μM + Chk1i 1 nM	ATMi 3 μM + Chk1i 3 nM	ATMi 3 μM + Chk1i 10 nM
HT29	CI	0.74	0.78	0.97	0.90
	FA	0.29	0.29	0.28	0.44
		ATMi 10 μM + Chk1i 0.03 μM	ATMi 10 μM + Chk1i 0.1 μM	ATMi 10 μM + Chk1i 0.3 μM	ATMi 10 μM + Chk1i 1 μM
SF-TY	CI	0.70	0.82	1.50	0.68
	FA	0.29	0.29	0.28	0.44

CI, combination index; FA, fraction affected.
